# Dimethyl-2-oxoglutarate but not antioxidants prevents glucose hypometabolism induced neural cell death: implications in the pathogenesis and therapy of Alzheimer's disease

**DOI:** 10.1016/j.bbrep.2025.102150

**Published:** 2025-07-12

**Authors:** Aman Chauhan, Karanpreet Bhutani, Aritri Bir, Ajay Singh, Sankha Shubhra Chakrabarti, Adesh K. Saini, Sasanka Chakrabarti, Arindam Ghosh

**Affiliations:** a Department of Biochemistry, Maharishi Markandeshwar Institute of Medical Sciences & Research, Maharishi Markandeshwar (Deemed to be University), Mullana, Ambala, Haryana, India; b Department of Biochemistry, Dr B. C. Roy Multi-Speciality Medical Research Centre, Indian Institute of Technology, Kharagpur, West Bengal, India; c Indian Institute of Technology, Jammu, Jammu & Kashmir, India; d Department of Geriatric Medicine, Institute of Medical Sciences, Banaras Hindu University, Varanasi, Uttar Pradesh, India; e Department of Bio-sciences & Technology, MMEC, Maharishi Markandeshwar (Deemed to be University), Mullana, Ambala, Haryana, India

**Keywords:** Antioxidant, Mitochondrial dysfunction, Glucose hypometabolism, GLUT inhibitor, Reactive oxygen species

## Abstract

Cerebral glucose hypometabolism is a cardinal molecular signature of Alzheimer's disease, and its role in the pathogenesis of this disorder is under intensive study in both animal and cell-based models. In the current study, we exposed SH-SY5Y cells (human neuroblastoma cell line) over a period of 48 h to DRB18, an inhibitor of multiple glucose transporters, in different concentrations to develop a state of glucose hypometabolism. Under this metabolic insult, in SH-SY5Y cells a profound dose-dependent neural cell death, an increased production of reactive oxygen radicals, mitochondrial membrane depolarization and a depletion of cellular ATP content were noted; these effects were not prevented by lipid-soluble novel antioxidants such as ferrostatin-1 and liproxstatin-1 or by a general water-soluble antioxidant like N-acetylcysteine. However, dimethyl-2-oxoglutarate, the cell-permeable analogue of 2-oxoglutarate (α-ketoglutarate) which can serve as an alternative fuel during glucose hypometabolism partially prevented both mitochondrial impairments and neural cell death. Thus, dimethyl-2-oxoglutarate may be explored further as a potential neuroprotective compound for Alzheimer's disease, and its effect on amyloid beta metabolism and homeostasis should be examined under glucose hypometabolic stress.

## Highlights

• DRB18, the GLUT-inhibitor, induces loss of cell viability in SH-SY5Y cells.
• DRB18 increases reactive oxygen species production in SH-SY5Y cells.
• Antioxidants cannot prevent DRB18 induced cell death.
• DRB18 also causes mitochondrial impairment in SH-SY5Y cells.
• All DRB18 effects in SH-SY5Y cells are prevented by dimethyl-2-oxoglutarate.

## Graphical abstract

**Figure:**
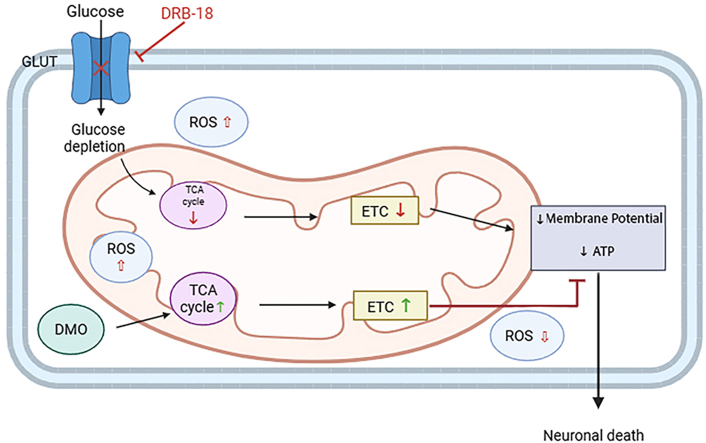

## 1 Introduction

The pathogenesis of sporadic Alzheimer's disease (AD) is complex, but Amyloid Cascade Hypothesis has occupied the centre stage for several decades in this regard; the mainstay of this hypothesis is the multitude of toxic actions of amyloid beta 42 derived from the amyloid precursor protein (APP) leading to neuronal degenerations in several parts of the brain concerned with cognition and memory [1]. Several alternative hypotheses also attempt to explain the pathogenesis of AD primarily from a metabolic angle, and mitochondrial dysfunction, decreased cerebral glucose utilization, brain insulin resistance and others are some of the important metabolic alterations implicated in AD pathogenesis [[2], [3], [4], [5]]. In fact, the National Institute on Aging – Alzheimer's Association (NIA-AA) research framework has included decreased cerebral glucose utilization as measured by ^18^F-2-deoxyglucose positron emission tomography (PET) -imaging as an in vivo marker of neuronal damage in AD [6]. However, the detailed mechanisms of decreased cerebral glucose utilization and its involvement in the pathogenesis of AD are yet to be elucidated. In this context both animal and cellular models of AD based on glucose hypometabolic stress and brain insulin resistance are being investigated, and many molecular signatures of AD have been replicated in cell-based glucose deprivation models [7,8]. For cell-based models of glucose deprivation-induced AD like pathology, mouse N2A neuroblastoma cells, hippocampal neural stem cells, human iPSC derived neurons, rat PC12 cells, SH-SY5Y cells (human neuroblastoma cell line) and several others have been used; glucose hypometabolism has been induced by exposure to streptozotocin or by depleting the glucose content of the media very significantly [7]. Recently, one group has reported in several publications that glyceraldehyde, an inhibitor of the glycolytic pathway, can cause neural cell death along with mitochondrial impairment, increased reactive oxygen species (ROS) production, Ca^2+^ dysregulation and increased amyloid beta 42 (Aβ42) formation in neuronally differentiated SH-SY5Y cells or primary cultures of rat cortical neurons [9,10]. Likewise in our lab it has been demonstrated that the glucose transporter (GLUT) inhibitor WZB117 which can inhibit GLUT-1, GLUT-3 and GLUT-4 with varying degrees of efficacy, can cause death of SH-SY5Y cells accompanied by mitochondrial impairments, increased production of ROS, increased formation of Aβ42 and enhanced activity of beta-site cleavage enzyme 1 (BACE1) [11]. The rationale of using GLUT-inhibitors to recreate the pathology of AD in this cell based model is justified because multiple post-mortem studies on the AD brain indicated decreased levels of GLUT1, GLUT3 and GLUT4 as summarized in a recent review [5]. Interestingly, in this model of neurodegeneration the ketone body β-hydroxybutyrate, acting as an alternative metabolic fuel for the cell has been shown to prevent the deleterious actions of WZB117 [11]. We thought it prudent, therefore, to explore newer aspects of the GLUT-inhibitor induced model of neurodegeneration by exposing SH-SY5Y cells to another pan-GLUT inhibitor, DRB18. In particular, we wanted to verify if increased ROS production in SH-SY5Y cells induced by glucose hypometabolic stress is pivotal to cellular death and further to identify other alternative fuels capable of preventing the effects of GLUT-inhibition.

## 2 Methods

### 2.1 Treatment protocol for cells

SH-SY5Y cells (NCCS, India) were maintained in a medium containing DMEM (Gibco, USA, Cat No. 12100-046) and Ham's F6 (Gibco, USA, Cat No. 21700-026) in the ratio of 2:1 (v/v); the medium was enriched with 10 % fetal bovine serum (Sigma, USA, Cat No. F7524) with added antibiotics (penicillin and streptomycin, 50 units/ml and 50 μg/ml respectively) and an anti-fungal agent (amphotericin B, 2.5 μg/ml). The cells were grown in 25 cm^2^ tissue culture flasks or multi-well plates and maintained in a CO_2_ incubator at 37^0^C under 95 % air and 5 % CO_2_. For experimental purposes, the cells were either untreated (control) or treated with the GLUT-inhibitor DRB18 (MedChemExpress, USA, Cat No. HY-145963) with varying concentrations (5–40 μM) for 48 h at 37^0^ C. Additionally for some experiments, DRB18 treated cells were co-treated with any of the other compounds such as ferrostatin-1 (1 μM, Sigma, USA, Cat No. SML0583), liproxstatin-1 (1 μM, Sigma, USA, Cat No. SML1414), N-acetylcysteine (2.5 mM, Sigma, USA, Cat No. A9165), pyruvate (5 mM, SRL, India, Cat No. 23569), succinate (5 mM SRL, India, Cat No. 87578), glutamate (5 mM, SRL, India, Cat No. 23229) and dimethyl-2-oxoglutarate or DMO (5 mM, Sigma, USA, 349631). After the incubation, SH-SY5Y cells were analysed for the loss of cell viability, ROS production, mitochondrial membrane depolarization and cellular ATP content. For each parameter the experiments were repeated several times (the number of observations mentioned in the legends to figures) with independent cell passages at different times.

### 2.2 Measurement of cell viability

The cell viability was measured by staining with Trypan blue using an automated counter (Countess 3FL, Thermo Fisher Scientific, USA) and cross-verified by manual counting as published earlier [11]. Additionally, with cells grown on multi-well plates, LDH leaking out of the dead cells into the medium was measured spectrophotometrically (Shimadzu, Japan) by monitoring NADH (SRL, India, Cat No. 54941) oxidation at 340 nm using pyruvate as the substrate; the enzyme activity (μmole NADH oxidized per min) was normalized to the cellular protein content of each well [12].

### 2.3 Measurement of ROS

For the measurement of cellular ROS, 2′,7′-dichlorodihydrofluorescein diacetate (H_2_DCFDA, Sigma, USA, Cat No. D6883) was used as a fluorogenic probe. H_2_DCFDA is first de-esterified within the cells, and the resulting DCFH_2_ reacts with multiple members of ROS to produce a fluorescent product. The measurement was carried out in control and variously treated cells in multi-well plates, and the fluorescence was recorded (λex 485 nm, λem 535 nm) in a multi-mode microplate reader (Molecular Devices, USA) as previously described [12].

### 2.4 Measurement of mitochondrial functions

The mitochondrial membrane potential was measured in cells grown in multi-well plates by using the fluorescent dye tetramethylrhodamine ethyl ester or TMRE (Thermo Fisher Scientific, USA, Cat No. T669) having λex 549 nm and λem 575 nm. This membrane permeable cationic fluorescent dye accumulates within the mitochondria, and in depolarized mitochondria the accumulation is less because of decreased electrochemical gradient. The procedure was described in details in our earlier publication [12]. The cellular ATP content was measured by the luciferin-luciferase based assay using a commercial kit (Sigma, USA, Cat No. FLAA); the assay was conducted in a multi-well plate and the luminescence was recorded on a multimode plate reader (Molecular Devices, USA) as detailed earlier [13]. The luminescence values were converted to ATP content using a calibration curve of pure ATP, further normalized to the protein content of each well and finally expressed as the percentage of the ATP content of control cells.

### 2.5 Protein estimation

The protein content of the samples was determined by bicinchoninic acid or BCA (Sigma, USA, Cat No. D8284) assay; the sample was solubilized by adding 1 % sodium dodecyl sulphate prior to the assay [12].

### 2.6 Statistical analysis

For statistical comparisons among more than two groups, one-way ANOVA with post-hoc Tukey's test was performed; the data were checked for normality by Shapiro-Wilk test before applying ANOVA. For comparisons among two groups, unpaired Student's *t*-test was performed. A p value < 0.05 was considered statistically significant. Results were expressed as the means ± SD of the number of independent observations indicated in the figure legends; individual data points are shown in column diagrams. GraphPad Prism software (version 8.4) was used for statistical analysis.

## 3 Results

**Fig. 1:**
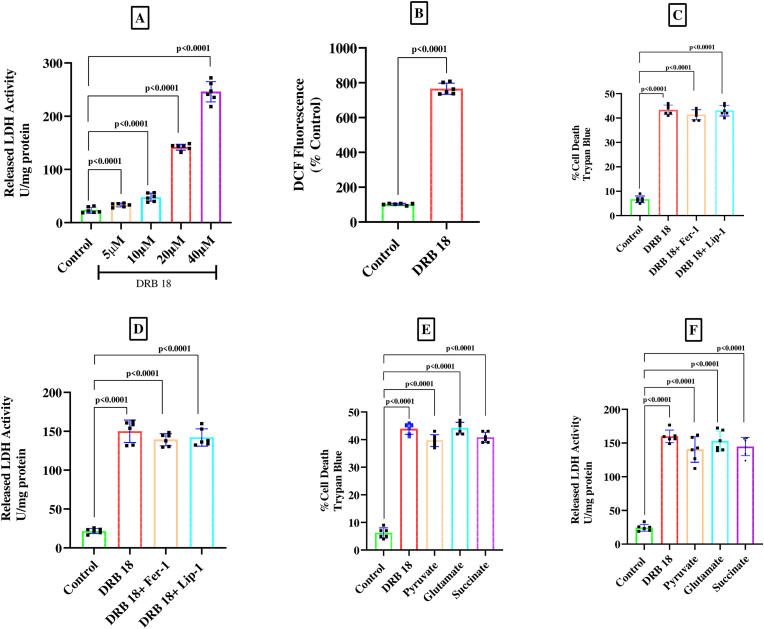
DRB18 induced death of SH-SY5Y cells SH-SY5Y cells were incubated for 48 h in CO_2_ incubator at 37^0^ C and subsequently analysed for cell death or ROS production; cells were untreated (control) or treated with various concentrations of DRB18 or DRB18 (20 μM) plus other additions such as ferrostatin-1 (Fer-1, 1 μM) or liproxstatin −1 (Lip-1, 1 μM) or pyruvate (5 mM) or succinate (5 mM) or glutamate (5 mM). A, D, F: Cell death measured by released LDH assay; B: ROS production by H_2_DCFDA assay; C, E: cell death by Trypan blue method. Values (means ± SD) are from 6 independent observations (biological replicates). One-way ANOVA was performed followed by post-hoc Tukey's test; p < 0.05 was considered statistically significant.

Results presented in Fig. 1A show that DRB18 caused a dose-dependent death of SH-SY5Y cells, and with 20 μM and 40 μM of DRB18 the cell death was increased by around 6-fold and 10-fold respectively (one-way ANOVA, F_4,25_ = 543.8, p < 0.0001; post-hoc Tukey's test, significant cell death with respect to control at 10 μM (p = 0.0024), 20 μM (p < 0.0001) and 40 μM (p < 0.0001) of DRB18). The production of ROS in SH-SY5Y cells was increased markedly (by more than 7-fold) (p < 0.0001, unpaired *t*-test) following treatment with DRB18 as shown in Fig. 1B. However, novel lipid-soluble antioxidants ferrostatin-1 and liproxstatin-1 failed to prevent the pronounced cell death caused by DRB 18 as measured by Trypan blue exclusion and LDH release assays respectively (Fig. 1C and D). The general water-soluble antioxidant N-acetylcysteine also failed to prevent neural cell death caused by DRB18 (data not shown). Likewise, several alternative metabolic fuels such as pyruvate (5 mM), succinate (5 mM) and glutamate (5 mM) were also ineffective in preventing DRB18 induced death of SH-SY5Y cells (Fig. 1E and F). Statistical analysis was performed by one-way ANOVA [F_3,20_ = 523.4, p < 0.0001; F_3,20_ = 221.4, p < 0.0001; F_4,25_ = 398.8, p < 0.0001; F_4,25_ = 110.0, p < 0.0001 for Fig. 1C–D, Fig. 1E and F respectively]. Pot-hoc Tukey's test revealed significant effect of DRB18 (p < 0.0001) on cell death with respect to controls, but statistically non-significant protective effect of any of the compounds such as ferrostatin−1, liproxstatin-1, pyruvate, glutamate and succinate against DRB18 induced cell death (Fig. 1C–D, Fig. 1E and F).

**Fig. 2:**
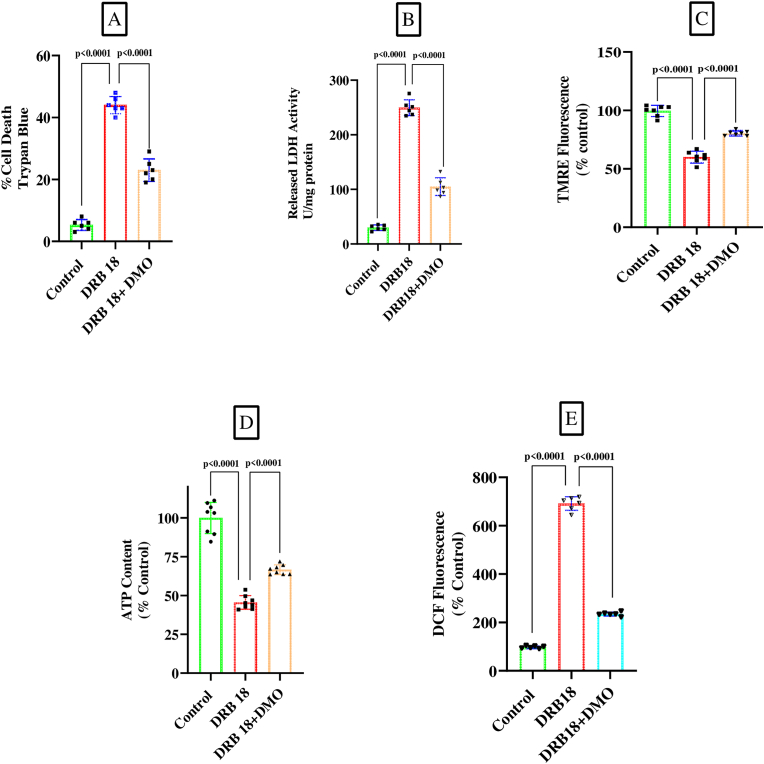
DMO prevents cell death and mitochondrial impairment in SH-SY5Y cells SH-SY5Y cells were untreated (control) or treated with DRB18 (20 μM) or DRB18 (20 μM) plus DMO (5 mM) for 48 h as described in the Experimental Procedures. Cell death was assessed by Trypan blue assay (A) or LDH activity released in the medium (B). Mitochondrial membrane potential was measured by TMRE assay (C), intra-cellular ATP content by luciferin-luciferase assay (D) and ROS by H_2_DCFDA assay (E). Values represent means ± SD, n = 6 for A and B; n = 7 for C, n = 8 for D, n = 6 for E (n represents the number of independent biological replicates) One-way ANOVA with Tukey's post-hoc test was performed; p < 0.05 was taken as significant.

The effect of DMO, a cell-permeable analogue of α-ketoglutarate, was examined on DRB18 induced cell death, ROS production and mitochondrial impairment. At a concentration of 5 mM, DMO partially, though markedly, prevented the cell death caused by DRB18 as observed by Trypan blue exclusion method or by measuring LDH activity leaked in the media (Fig. 2A and B). One-way ANOVA was used to analyse group differences (F_2,15_ = 282.6, p < 0.0001 for Fig. 2A and F_2,15_ = 449.7, p < 0.0001 for Fig. 2B); post-hoc Tukey's test indicated a significant difference in cell death between control and DRB18 treated cells (p < 0.0001) and statistically significant prevention of DRB18 effect by DMO (p < 0.0001) in both Fig. 2A and B. Results shown in Fig. 2C indicate that DRB18 caused a decrease in mitochondrial membrane potential by approximately 40 percent which was prevented conspicuously, though partially, by co-treatment with DMO. Likewise, the intra-cellular ATP content was lowered by nearly 55 percent in SH-SY5Y cells following exposure to DRB18 which was prevented noticeably but only partially by DMO (Fig. 2D); similarly DRB caused a very large increase in ROS production in SH-SY5Y cells which was markedly but incompletely inhibited by DMO (Fig. 2E). Statistical comparisons among different groups were made by one-way ANOVA (F_2,18_ = 162.0, p < 0.0001 for Fig. 2C; F_2,21_ = 139.1, p < 0.0001 for Fig. 2D; F_2,15_ = 1965, p < 0.0001 for Fig. 2E). Post-hoc Tukey's test revealed a significant effect of DRB18 (p < 0.0001) on mitochondrial membrane potential, cellular ATP content and ROS production with respect to control, and a statistically significant prevention of DRB18 effect (p < 0.0001) by DMO in Fig. 2C–D and E.

## 4 Discussion

SH-SY5Y cells have been widely used to study APP and Aβ42 homeostasis and toxicity, tau phosphorylation, oxidative stress and neural cell death in the context of AD pathogenesis in different experimental models [[14], [15], [16]]. The role of glucose hypometabolism in AD neurodegeneration in model systems using SH-SY5Y cells is also being investigated [7]. The purpose of developing glucose hypometabolism based models of AD is not only to explore if decreased cerebral glucose utilization plays a dominant role in the disease pathogenesis, but also to identify potential disease-modifying drugs for this disorder [5,7]. In our earlier study we observed that the GLUT inhibitor WZB117 caused very significant loss of cell viability with marked increase in ROS production [11]. Since ROS have been linked with multiple regulated cell death pathways including ferroptosis, we have attempted to verify in the current study if the pan-GLUT inhibitor DRB18 could cause neural cell death through enhanced production of ROS. The increased production of ROS in SH-SY5Y cells following DRB18 exposure as seen in this study may be attributed to several factors, but a decreased availability of reduced glutathione because of a diminished pool of NADPH is likely to be a major mechanism. The diminished intracellular NADPH level from the pan-GLUT inhibition can arise in several ways. For example, a decreased utilization of glucose may lead to diminished supply of glucose-6-phosphate for the Hexose Monophosphate Shunt with a consequent decrease in cytosolic NADPH. Likewise, a reduced level of the TCA cycle intermediate isocitrate because of decreased glucose metabolism, may lower the mitochondrial NADPH synthesized by isocitrate dehydrogenase isoenzyme (IDH2) located in the mitochondrial matrix [17]. A third mechanism may involve mitochondrial nicotinamide nucleotide transhydrogenase converting NADPH to NADP+ and generating NADH from the accumulated NAD+ in DRB18 treated SH-SY5Y cells [18]. Furthermore, metabolic re-programing leading to the use of other fuels such as fatty acids by SH-SY5Y cells may lead to increased ROS production [19]. Thus, multiple mechanisms may contribute to the ROS accumulation after DRB18 treatment, but it is surprising that this build-up of intracellular ROS may not lead to cell death because the novel lipid soluble antioxidants ferrostatin-1 and liproxstatin-1 failed to inhibit the latter; the water-soluble general antioxidant N-acetylcysteine was also ineffective in preventing DRB18 induced cell death (data not presented). This surprising result needs to be probed further in view of the fact that oxidative stress is considered as an early pathological event in the brain in clinical AD cases or transgenic AD models [20,21].

In our earlier study it was observed that β-hydroxybutyrate, a ketone body, could serve as an alternative fuel and significantly prevent the mitochondrial bioenergetic impairment and death of SH-SY5Y cells exposed to the GLUT-inhibitor WZB117. Thus, we thought it prudent to examine if other alternative fuels could prevent the loss of cell viability and mitochondrial impairment in SH-SY5Y cells after exposure to DRB18. However, pyruvate, succinate and glutamate failed to prevent DRB18 induced neural cell death. Dimethy-2-oxoglutarate, the cell-permeable analogue of α-ketoglutarate, however, significantly, though incompletely, prevented the cell death and impairment of mitochondrial bioenergetic functions. The failure of succinate and pyruvate to serve as alternative fuels is possibly related to their limited uptake in the SH-SY5Y cells or stability in aqueous medium [[22], [23], [24]]. On the other hand, cell-permeable DMO is presumably converted to 2-oxoglutarate within the cells which serves as an anaplerotic reaction of the TCA cycle; the phenomenon results in the production of enough reducing equivalents for the mitochondrial electron transport chain (ETC) leading to the partial but significant recovery of mitochondrial membrane potential, ATP synthesis and eventual cell viability. Expectedly, DMO also decreases the ROS production following DRB18 treatment, but this may not be of a significant consequence with respect to cell viability because antioxidants are ineffective in preventing cell death in the current study. However, the reason for the failure of glutamate to provide protection against DRB 18 induced cell death is not clear; at the concentration used in this study glutamate had no direct toxic effect on cell viability (data not shown).

Overall, this brief study has primarily provided some clues in elucidating the role of glucose hypometabolism in triggering mitochondrial impairment and neural death in the context of AD pathogenesis. However, it will not be out of place to discuss a little about the therapeutic potential of DMO in AD. Our earlier study identified the ketone body β-hydroxybutyrate as effective in preventing glucose hypometabolism induced mitochondrial impairment, neural cell death and altered amyloid beta homeostasis [11]. Interestingly, ketogenic diets which increase the circulating levels of ketone bodies such as β-hydroxybutyrate have been proven to be beneficial in improving cognitive deficits in AD as recently reported in a systematic review and meta-analysis [25]. A randomized cross-over study has also reported similar beneficial effects in AD [26]. The ketogenic diets can refuel the energy metabolism of the AD brain by providing acetyl-CoA to the TCA cycle in the face of decreased cerebral glucose utilization; however ketogenic diets have several adverse effects including hypoglycaemia [25]. Thus, it may be questioned whether lower carbohydrate content of a ketogenic diet formulation with associated risk of hypoglycemia is a viable option to provide energy to the brain when the organ already has an impaired glucose utilization in AD. On the other hand, the current results indicate that DMO could be another alternative energy substrate to the brain and a potential neuroprotective compound in the context of decreased cerebral glucose metabolism in AD; however, the effects of DRB18 in altering other features of AD such as amyloid beta accumulation or increased tau phosphorylation and the possible reversal of such effects by DMO are to be examined thoroughly.

## CRediT authorship contribution statement

**Aman Chauhan:** Writing – original draft, Methodology, Formal analysis. **Karanpreet Bhutani:** Methodology, Investigation. **Aritri Bir:** Writing – original draft, Conceptualization. **Ajay Singh:** Methodology, Investigation. **Sankha Shubhra Chakrabarti:** Writing – review & editing, Conceptualization. **Adesh K. Saini:** Writing – original draft. **Sasanka Chakrabarti:** Writing – review & editing, Validation, Methodology, Funding acquisition, Conceptualization. **Arindam Ghosh:** Writing – review & editing, Visualization, Validation, Methodology, Funding acquisition, Conceptualization.

## Funding

The authors wish to thank the authority of Maharishi Markandeshwar (Deemed to be University) for funding and administrative support.

The authors gratefully acknowledge AG for partial financial support of the experiments through the intramural grant from 10.13039/501100008984IIT Kharagpur (Grant No. IIT/SRIC/FSRGI2023/17).

## Declaration of competing interest

The authors declare that they have no known competing financial interests or personal relationships that could have appeared to influence the work reported in this paper.

## Data Availability

Data will be available from the corresponding author on reasonable request.
